# Late-Onset Hematoma Due to Bleeding of a Small Branch of the Lateral Circumflex Femoral Artery Following Proximal Femur Intramedullary Nailing

**DOI:** 10.7759/cureus.23513

**Published:** 2022-03-26

**Authors:** Ioannis Papaioannou, Georgia Pantazidou, Thomas Repantis, Vasileios K Mousafeiris, Nektaria Kalyva

**Affiliations:** 1 Orthopedics, General Hospital of Patras, Patras, GRC; 2 Otolaryngology - Head and Neck Surgery, General Hospital of Patras, Patras, GRC; 3 Pediatrics, University Hospital of Patras, Patras, GRC

**Keywords:** anticoagulants, bleeding, intramedullary locked nail, lateral circumflex femoral artery, hematoma

## Abstract

Intramedullary nailing of proximal femur fracture is not deprived of complications, although vascular complications are very rare and a high index of suspicion is required for timely diagnosis. This case report describes how a late-onset hematoma formation and bleeding of a small branch of the lateral circumflex femoral artery can complicate intramedullary nailing after a pertrochanteric fracture. To the best of our knowledge, this complication has never been reported and should be considered among the possible vascular complications of intramedullary nailing. Orthopedic surgeons should be aware of the vascular complications that can occur even with late-onset presentation and even from small vessels, while administration of anticoagulants is an aggravating factor. Elderly patients with proximal femur fractures are more susceptible to vascular injury due to the structure of their vessels and the vicinity of the fracture to the arterial supply of the hip.

## Introduction

Fragility hip fracture is the most common orthopedic injury among elderly patients and represents a huge public health issue with significant socioeconomic impact [1]. Surgical treatment of trochanteric hip fractures includes extramedullary implants, intramedullary nails, and arthroplasty. During the last 15 years, intramedullary nailing gained popularity among orthopedic surgeons and became the implant of choice for the majority of extracapsular hip fractures [2]. Intramedullary nailing is not deprived of complications, although vascular complications are very rare and require a high index of suspicion for timely diagnosis. These complications are potentially limb and life-threatening. The most common vascular injury concerns the profunda femoris artery with many reports in the literature [3-5]. The aim of this study is to appose how a late-onset hematoma formation and bleeding of a small branch of the lateral circumflex femoral artery can complicate intramedullary nailing after a pertrochanteric fracture and describe the proper management of the vascular complications. To the best of our knowledge, this complication has never been reported and should be considered among the possible vascular complications of intramedullary nailing.

## Case presentation

**Figure 1:**
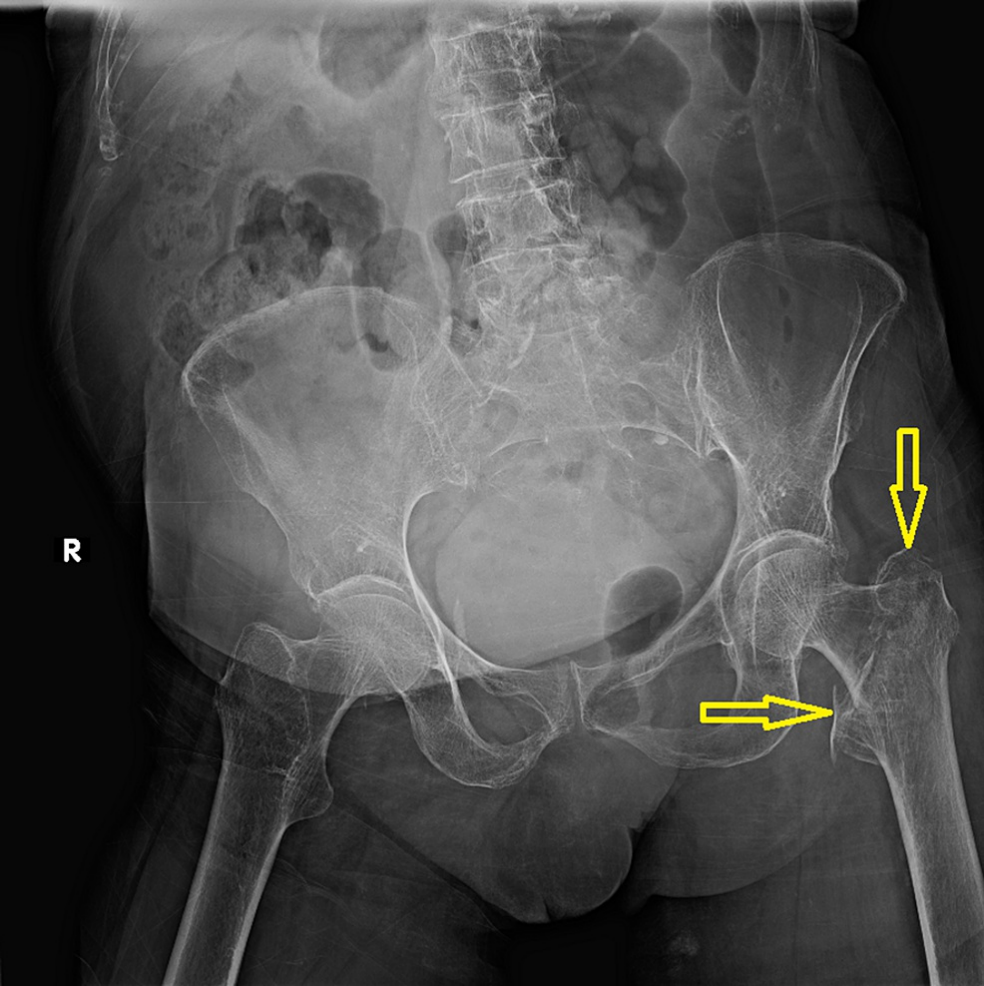
Anteroposterior plain radiogram of the hip and pelvis on admission to the emergency department. Yellow arrows demonstrate the fracture line of the left proximal femur.

An 84-year-old female patient was admitted to our emergency department after a ground-level fall. She reported landing directly on her left side and was unable to ambulate. Clinical examination demonstrated shortened and externally rotated leg accompanied with worsening groin pain with any manipulation of the left leg. She was unable to perform a straight leg raise, while there was no neurovascular deficit. The patient’s medical history included type 2 diabetes mellitus (insulin therapy) and atrial fibrillation treated with apixaban. Apixaban was stopped for 56 hours before surgery, while preoperative blood tests were within normal limits. Radiographic evaluation revealed a minimal displaced intertrochanteric fracture of the left hip, classified as 31 A1 according to the AO/OTA (Arbeitsgemeinschaft für Osteosynthesefragen/Orthopaedic Trauma Association) classification (Figure 1).

**Figure 2:**
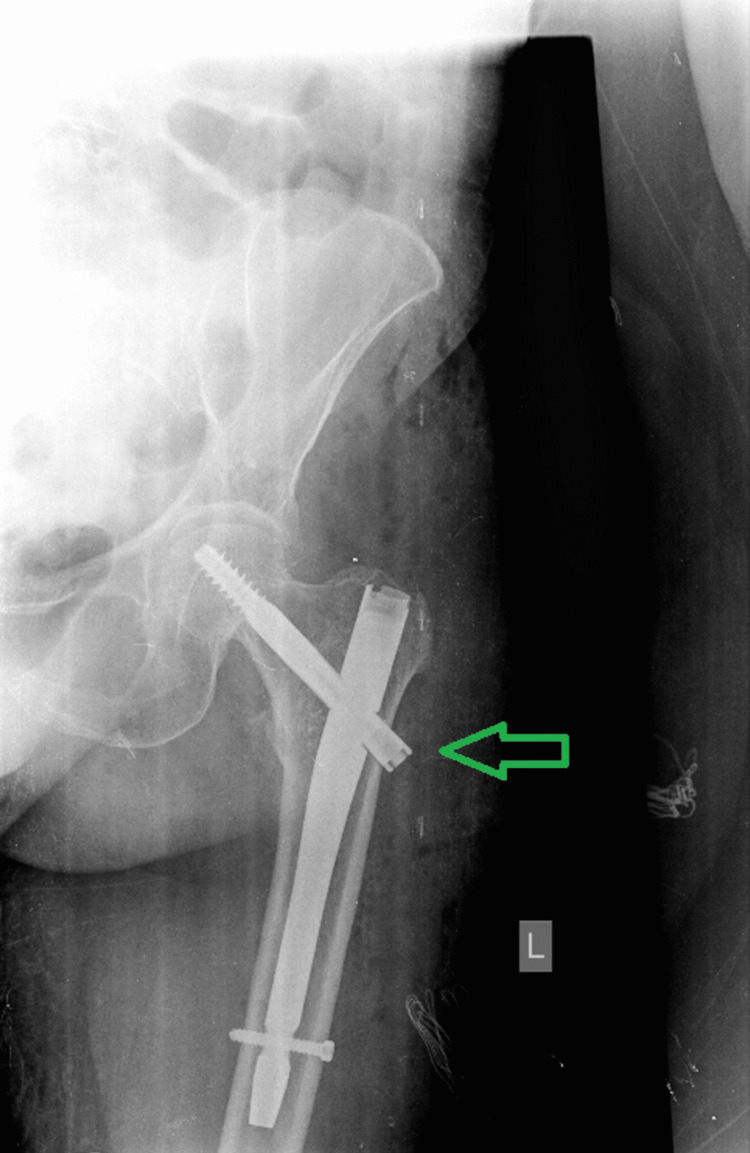
Postoperative anteroposterior plain radiogram of the left hip and proximal femur. The green arrow demonstrates the Gamma 3 nail with adequate fracture reduction and a very satisfying outcome.

A short Gamma 3 nail (Trochanteric Nail 180, Stryker Trauma GmbH, Schönkirchen, Germany) was implanted two days after admission and the patient had an uneventful recovery (Figure 2).

**Figure 3:**
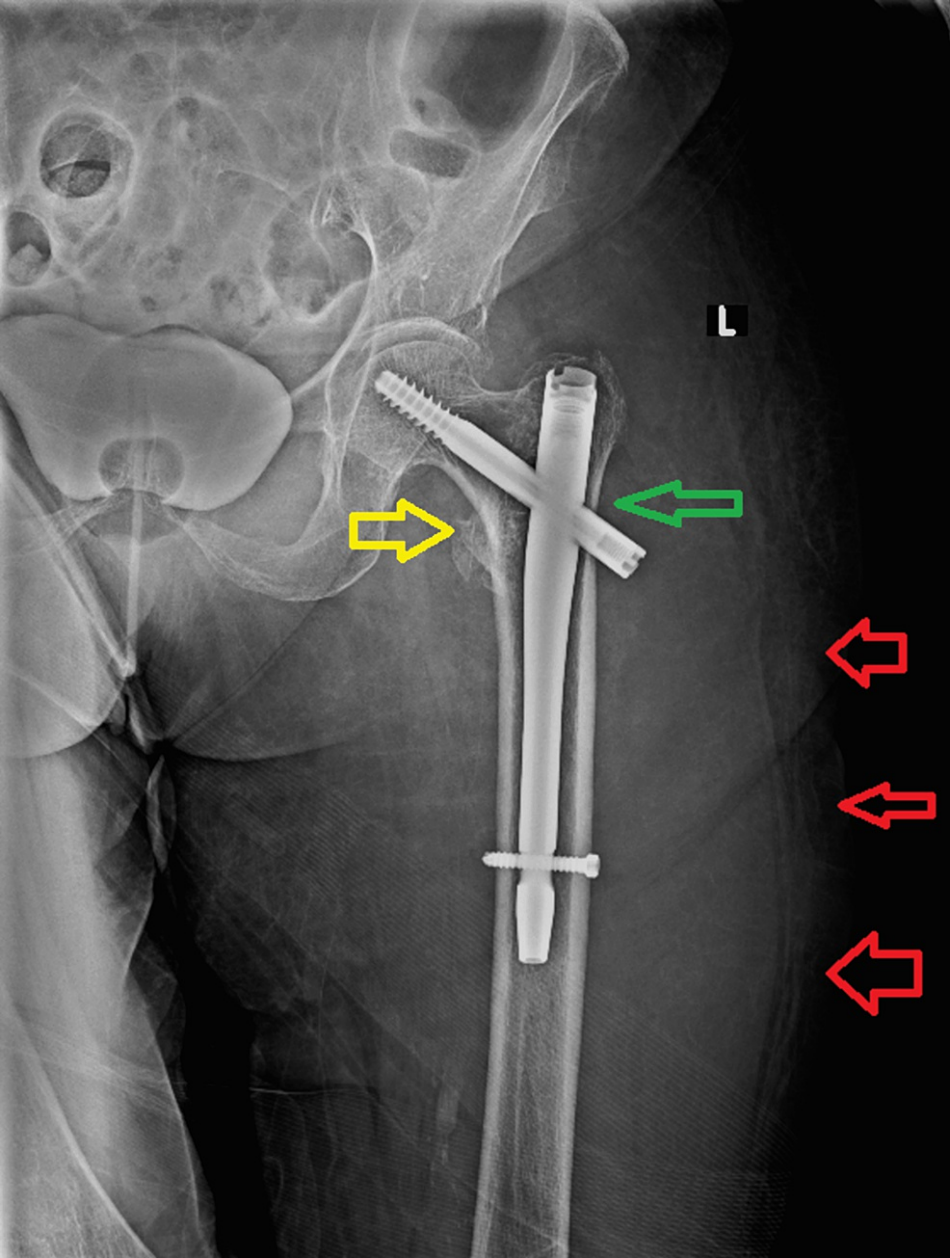
Anteroposterior plain radiogram of the left hip and proximal femur of our patient on the second admission to the emergency department. The implant was in the previous correct position, while the yellow arrow demonstrates the compression of the fracture line and the green arrow shows the sliding of the lag screw. Red arrows indicate the excessive edema of the thigh.

Three days postoperatively, the patient was hemodynamically stable with a hematocrit of 31% and hemoglobin of 10.3 g/dL, stable for three days, and was discharged. Twenty-five days after surgery, she was admitted again to our emergency department due to significant edema of the left thigh accompanied with remarkable pain, weakness, and inability to bear weight. The radiographic evaluation was normal, without any displacement of the implant (Figure 3).

**Figure 4:**
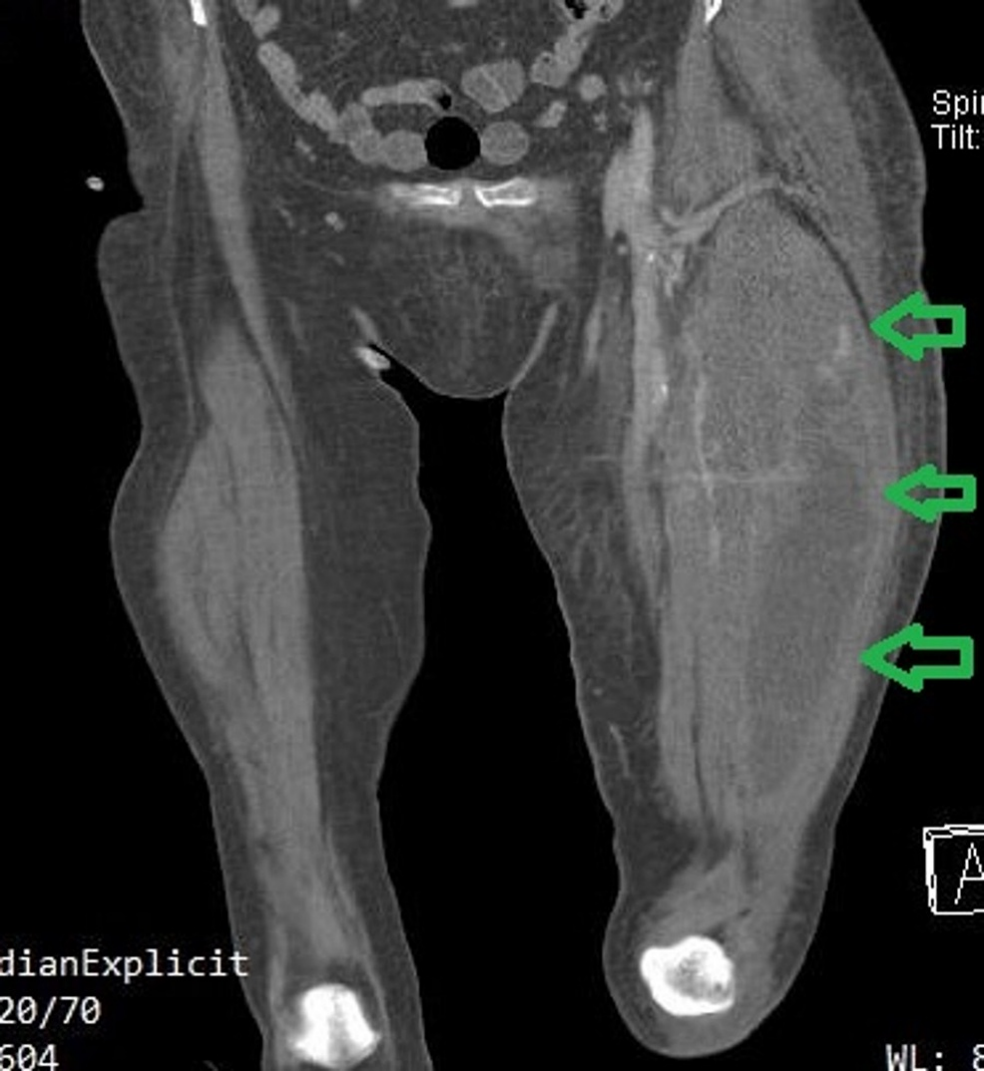
Coronal computed tomography angiography image indicates the huge hematoma formation of the left thigh (green arrows).

**Figure 5:**
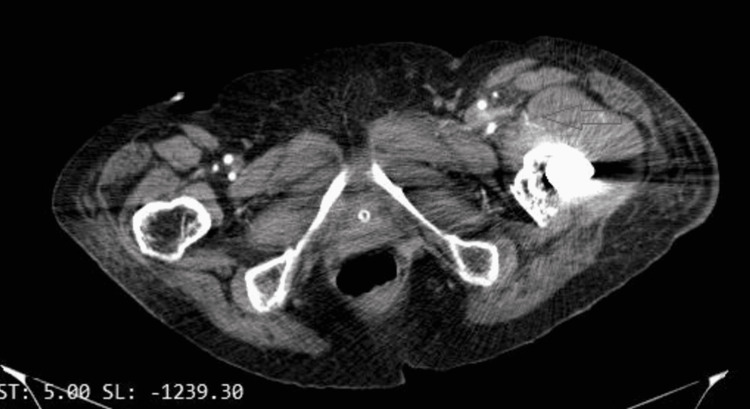
Axial computed tomography angiography image demonstrates the small extravasation (red arrow).

The patient’s hemoglobin was found to be 6.9 g/dL and she underwent a blood transfusion. Computed tomography angiography (CTA) revealed a large hematoma on the lateral side of the thigh (Figure 4) and active small extravasation (Figure 5). Once the diagnosis was settled, apixaban was stopped until the bleeding is completely controlled and the patient remains hemodynamically stable.

**Figure 6:**
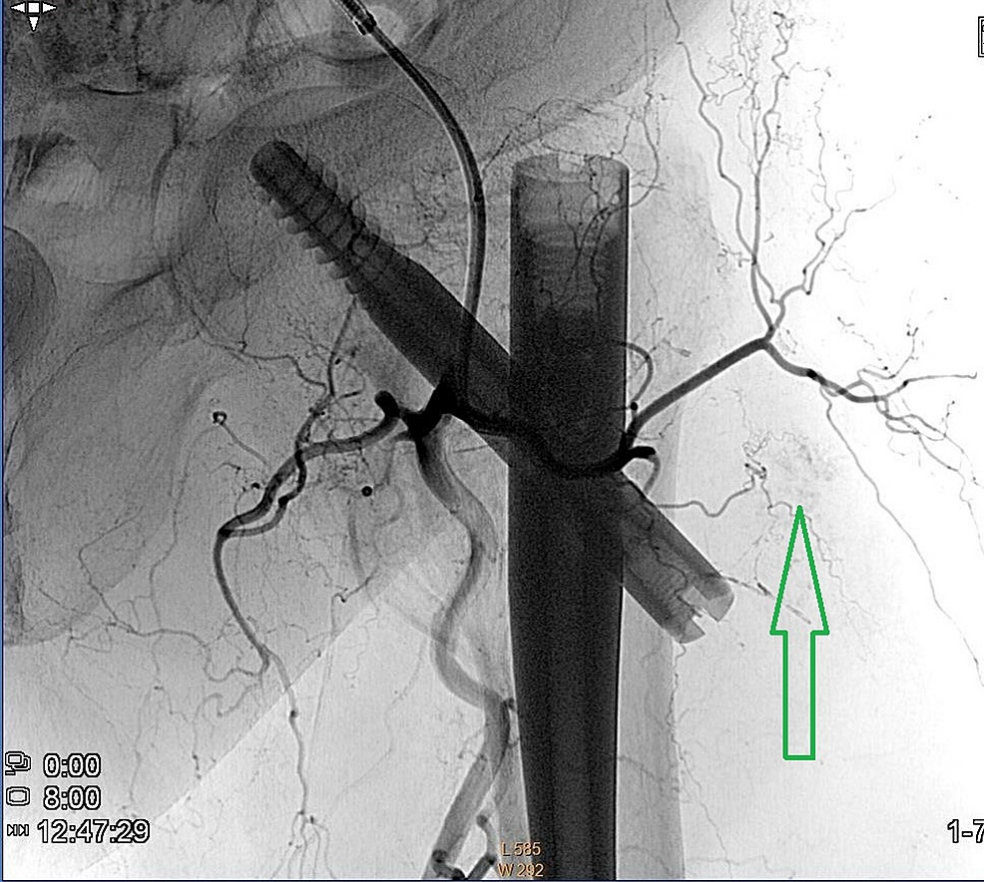
Coronal conventional angiography image of the left hip and proximal femur demonstrates the arterial flow to the hip, while the green arrow demonstrates active bleeding.

**Figure 7:**
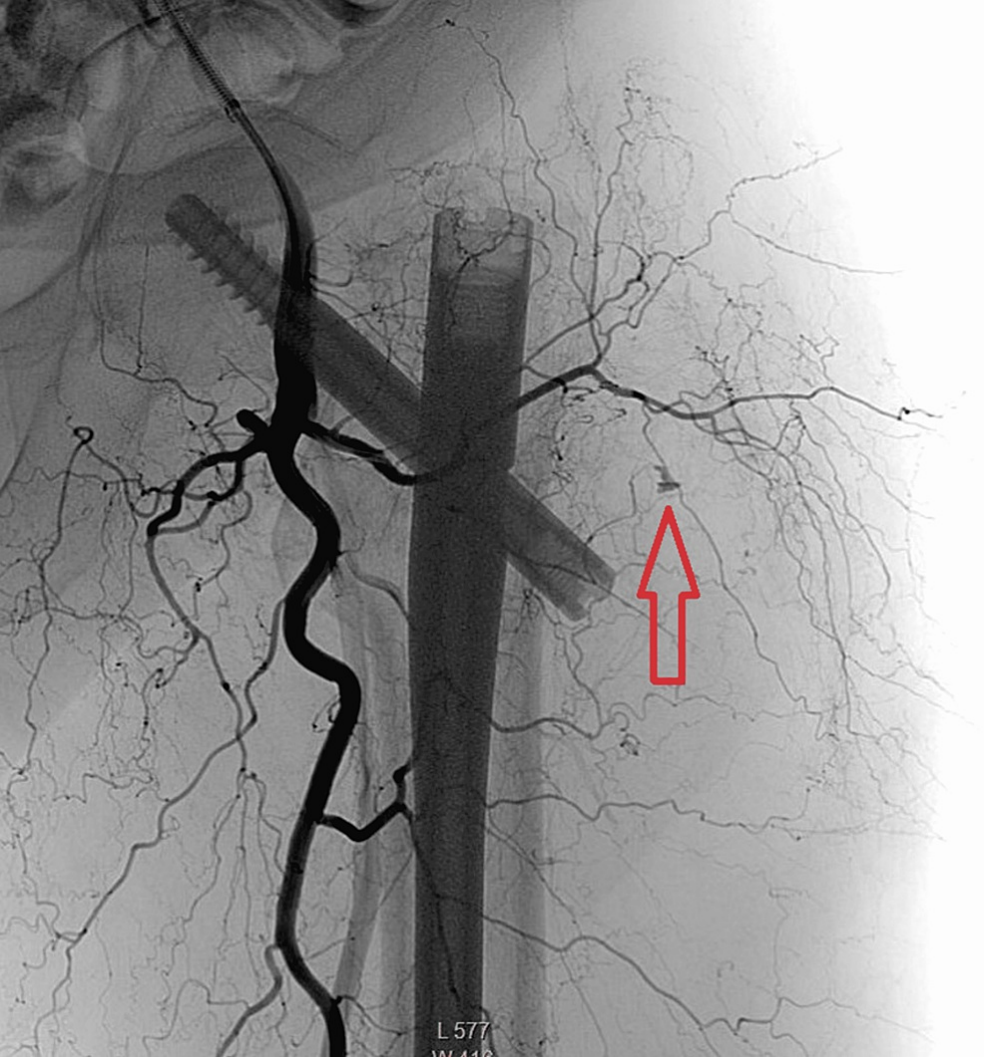
Coronal conventional angiography image of the left hip and proximal femur demonstrates the coil embolization and stoppage of bleeding (red arrow).

The patient was referred to the invasive radiology department and underwent conventional angiography. A small branch of the lateral circumflex femoral artery (branch of the deep femoral artery) was found with active bleeding (Figure 6). Coil embolization was performed and the bleeding was stopped (Figure 7).

The edema of the thigh was resolved gradually, hemoglobin was stabilized to previous levels, and the patient returned to the rehabilitation unit three days after embolization. Apixaban administration was initiated 48 hours after coil embolization. The fracture was healed three months after the initial hip surgery and our patient returned to her daily routine six months later.

## Discussion

Arterial damage during fixation of intertrochanteric hip fractures is a very rare complication [3], with a reported incidence of 0.2% [6]. However, if present, these complications can cause limb dysfunction or even become life-threatening. The most common vascular complication concerns the deep femoral artery, followed by superficial femoris, superior gluteal, and inferior epigastric arteries [3]. Vascular injuries can be hemorrhagic or thrombotic, acute with active bleeding, or delayed with pseudoaneurysm formation [6]. Neurovascular structures were susceptible to iatrogenic intraoperative injury during percutaneous insertion of implants such as pins, drill bits, screw tips, and blades, or by a displaced bone fragment at the time of fracture or during fixation.

The mechanisms of vascular injury are mainly iatrogenic due to the inappropriate use of surgical instruments. Iatrogenic vascular injury has also been observed due to forceful traction and during maneuvers to reduce lesser trochanter fractures. Most of the spontaneous non-iatrogenic vascular injury mechanisms concern the fragment of lesser trochanter [6].

The most common vascular injury during fixation of a hip fracture is the pseudoaneurysm formation of the profunda femoris artery [6], with many reports in the literature [4,5]. Pseudoaneurysms or false aneurysms are caused by partial arterial vessel damage with subsequent formation of a hematoma, which is in contact with the arterial lumen [4]. The first and historical case report was published in 1964 by Dameron [7]. The treatment of choice for deep femoral artery pseudoaneurysm is the coil embolization or covered stent insertion, while other treatment options are ultrasound-guided compression, percutaneous ultrasound-guided thrombin injection, percutaneous ultrasound-guided collagen injection, and open surgery [6].

Damage to the profunda femoris artery or its perforating branches is usually reported due to their close relationship to the femur in the subtrochanteric region, while injury of the superficial femoral artery or its branches is much less frequent [4]. Even rarer is the injury of the lateral circumflex femoral artery and its branches. The literature review revealed only five cases with vascular injury of the lateral circumflex femoral artery. Four of them concern pseudoaneurysm formation after fixation of femoral neck fracture [8-11] and only one concerns an intertrochanteric fracture [12]. Vascular injury in the cases with femoral neck fracture was caused after the fracture fixation due to iatrogenic intervention, while in the intertrochanteric fracture case, the injury of the lateral circumflex femoral artery was caused by the lesser trochanter fragment.

To our knowledge, the lateral circumflex femoral artery has never been reported to complicate a nail fixation of an intertrochanteric fracture. We strongly believe that the damage to this small vessel was caused during the attempts for proper awl insertion by our young resident. We encourage young orthopedic surgeons to check the awl position with an image intensifier before insertion or alternatively to use a guidewire to identify the proper entry point and then insert the awl through the wire. Additionally, the apixaban re-administration three days after surgery probably worsened the bleeding and contributed to the hematoma formation.

The atherosclerotic, fragile and more rigid low-flow arteries of elderly patients are more susceptible to injury from surgical instruments or during intraoperative maneuvers [6].

Timely diagnosis is of paramount importance and can lead to proper treatment. Misdiagnosis can lead to limb ischemia and can be potentially life-threatening [6]. Compartment syndrome is another possible complication of subacute vascular injury and should also be considered [13]. There is debate in the current literature concerning the appropriate diagnostic algorithm and treatment of vascular injuries after internal fixation of proximal femur fractures [6]. The etiology of this debate is the wide spectrum of vascular injury, which varies from vessel laceration with massive bleeding to pseudoaneurysm formation accompanied by subacute bleeding and hematoma formation. In any case, if there is any fragment or implant that interferes with the injured vessel, it should be removed. Furthermore, minimally invasive intervention from radiologists or even extensive surgery from vascular surgeons is mandatory to restrict and stop bleeding. Despite the rarity of vascular injury after internal fixation of proximal femur fractures, the morbidity and mortality rates are 11.44% and 6.62%, respectively [6].

## Conclusions

Orthopedic surgeons should be aware that vascular complications can occur even with late-onset presentation and even from small vessels, while administration of anticoagulants is an aggravating factor. During the postoperative period, a significant reduction of hemoglobin accompanied with massive operative site swelling should alert orthopedic surgeons to diagnose this rare condition. Elderly patients with proximal femur fractures are more susceptible to vascular injury due to the structure of their vessels and due to the vicinity of the fracture to the arterial supply of the hip.
